# Renal Involvement in Non-Hodgkin Lymphoma: Proven by Renal Biopsy

**DOI:** 10.1371/journal.pone.0095190

**Published:** 2014-04-14

**Authors:** Shi-Jun Li, Hui-Ping Chen, Ying-Hua Chen, Li-hua Zhang, Yuan-Mao Tu, Zhi-hong Liu

**Affiliations:** National Clinical Research Center of Kidney Diseases, Jinling Hospital, Nanjing University of Medcine, Nanjing, China; Fondazione IRCCS Ospedale Maggiore Policlinico & Fondazione D'Amico per la Ricerca sulle Malattie Renali, Italy

## Abstract

**Aims:**

To determine the spectrum of renal lesions in patients with kidney involvement in non-Hodgkin's lymphoma (NHL) by renal biopsy.

**Methods:**

The clinical features and histological findings at the time of the renal biopsy were assessed for each patient.

**Results:**

We identified 20 patients with NHL and renal involvement, and the diagnosis of NHL was established following the kidney biopsy in 18 (90%) patients. The types of NHL include the following: chronic lymphocytic leukemia/small lymphocytic lymphoma (n = 8), diffuse large B-cell lymphoma (n = 4), T/NK cell lymphoma (n = 3), lymphoplasmacytic lymphoma (n = 2), cutaneous T-cell lymphoma (n = 1), mucosa-associated lymphoid tissue lymphoma (n = 1) and mantle cell lymphoma (n = 1). All presented with proteinuria, and 15 patients had impaired renal function. The pathological findings included (1) membranoproliferative glomerulonephritis-like pattern in seven patients; (2) crescent glomerulonephritis in four; (3) minimal-change disease in three, and glomeruli without specific pathological abnormalities in three; (4) intraglomerular large B-cell lymphoma in one; (5) intracapillary monoclonal IgM deposits in one; (6) primary diffuse large B-cell lymphoma of the kidneys in one; and (7) lymphoma infiltration of the kidney in eight patients.

**Conclusion:**

A wide spectrum of renal lesions can be observed in patients with NHL, and NHL may be first proven by renal biopsies for evaluation of kidney injury or proteinuria. Renal biopsy is necessary to establish the underlying cause of renal involvement in NHL.

## Introduction

Renal involvement in non-Hodgkin lymphoma (NHL) has been reported previously, including glomerulonephritis, acute kidney injury (AKI), and lymphoma infiltrating the kidney parenchyma [1]–[4]. Previous studies have shown that up to 10% of patients with NHL and lymphocytic leukemia may have kidney injury [5]. However, a limited number of cases of GN have been described in patients with NHL demonstrated by renal biopsy in the literature to date [6]–[8]. Considering that the morphology of glomerular injury in patients with lymphoma are often heterogeneous, the myriad of etiologies of renal injury due to NHL often present a diagnostic challenge to clinician. Here, we retrospectively analyzed the spectrum of renal lesions proven by renal biopsy in patients with NHL in single center, to better establish the relationship between renal injury and NHL.

## Materials and Methods

### Patient selection

We reviewed the renal pathology archives of the Research Institute of Nephrology at Nanjing University of Medicine from 2001 through 2012 and identified 20 patients with NHL and renal dysfunction of sufficient severity and/or proteinuria that a renal biopsy was obtained. The diagnosis of NHL was based on the 2008 WHO classification system [9]. This study was approved by the Ethical Committee of Nanjing University. According to the ethics committee recommendation, written consent was not required for this non interventional study. Patients or surrogates provided verbal informed consent prior to study inclusion. Verbal consent was obtained through a session of patient or family information explaining the study, its aims and the non interventional design. The consent was recorded in the medical chart of each patient. Patients or relatives had the opportunity to decline study participation at any time.

### Data collection

Baseline data at the time of renal biopsy were obtained from the medical records for all cases. The following data were collected: sex, age, clinical presentation, ultrasound of kidneys, extra renal presentations and relevant clinical history.

Clinical and laboratory data at the time of kidney biopsy were assessed for each patient. Cryoglobulinemia was detected by cold precipitation of serum samples from blood that had been collected and processed at 37°C. Proteinuria was defined as a urine protein level >0.4 g/24 h, and hematuria, assessed using light microscopy, was defined as a red blood cell count >10,000/ml in the urinary sediment. Nephrotic syndrome was defined as a urinary protein excretion >3.5 g/d and a serum albumin level <30 g/L. Impaired renal function was defined as a GFR<60 ml/min per 1.73 m^2^according to the Modification of Diet in Renal Disease (MDRD) formula. Acute kidney injury was defined according to the KIDIGO criteria.

### Renal biopsy studies

All patients underwent a percutaneous renal biopsy. No symptom perirenal haematoma and macroscopic haematuria have been observed in these patients. Each renal sample contained more than 10 glomeruli. The renal biopsy procedure was as follows: the samples were embedded in paraffin and sectioned at 2 µm, followed by hematoxylin-eosin, Masson, periodic acid-Schiff or periodic acid-silver methenamine (PASM) staining. For immunofluorescence (IF), the samples were sectioned at 3 µm using a cryostat, followed by use of a panel of FITC-conjugated rabbit anti-human antibodies to IgG, IgM, IgA, C3, C1q, and κ and λ light chains (polyclonal, Dako Corporation). These samples were also stained with Congo red. The intensity of the immunofluorescence staining was semiquantitatively scored on a scale of 0 to 2+. Immunophenotyping of the lymphomas was performed on frozen sections using the immunoperoxidase and avidin-biotin techniques. The phenotype of the cellular infiltrate was studied using biotinylated anti-CD4, -CD8, -CD20 and -CD68 antibodies (Dako) and visualized using a peroxidase-streptavidin conjugate. Electron microscopy was performed using a Hitachi 7500 electron microscope after routine sections were prepared from renal tissues, followed by double staining with uranyl acetate and lead citrate. Renal biopsies from all patients were reviewed by two renal pathologists.

## Results

### Clinical findings

The clinical characteristics of all 20 patients are summarized in Table 1. There were 17 men and 3 women aged 16 to 68 years at the time of renal biopsy (mean age 53 years). At the time of the renal biopsy, only 2 (10%) of 20 patients had an established diagnosis of NHL, the remaining 18 patients had renal biopsies performed before the diagnosis of the lymphoma. Six patients (30%) presented with recurring fever of unknown origin. Enlargement of the inguinal, axillary, submaxillary or supraclavicular lymph nodes were found in eight patients (40%). Computed tomography (CT) scans revealed numerous intumesced lymph nodes in the neck, mediastinal septum and retroperitoneal space in five patients (25%). CT revealed markedly enlarged kidneys bilaterally in nine (45%) patients, enlargement of the spleen in three patients. (See Table 1). The anatomic sites of sampling used for the primary diagnosis of the lymphoid neoplasm were the lymph nodes (n = 7), bone marrow (n = 6), gastrointestinal tract (n = 2), nasal cavity/sinus (n = 2), kidney (n = 2) and skin (n = 1).

**Table 1:** Clinical features of patients with NHL and renal involvement

Patient	Sex	Age	Type of lymphoma	Biopsy site	Clinical symptoms of lymphoma	Renal symptoms	Treatment	Patient outcome	Renal Outcome (Scr µmol/l)
**1**	M	16	T/NK	Nasal mucosa	Anemia, fever, nasal discharge	RPGN	EPOCH	Died (1 yr)	NA
**2**	F	38	T/NK	Nasal mucosa	Fever, nasal discharge	NS	Steroids	Died (2 mo)	NA
**3**	F	39	TCL	Lymph node	Fever, lymphadenectasis	proteinuria	CHOP	Died (2 mo)	NA
**4**	M	40	TCL	Skin	Skin rash	NS	Steroids	NA	NA
**5**	M	61	CLL/SLL	Bone marrow	Anemia, weakness	AKI	Chlorambucil	Died (2 mo)	NA
**6**	F	57	CLL/SLL	Lymph node	Anemia, lymphadenectasis	RPGN	Chlorambucil	NA	NA
**7**	M	64	CLL/SLL	Lymph node	Lymphadenectasis	RPGN	FC	Died (3 mo)	NA
**8**	M	55	CLL/SLL	Lymph node	Lymphadenectasis	RPGN	FC	Alive (3 yr)	dialysis
**9**	M	59	CLL/SLL	Bone marrow	Leucocytosis	RPGN	Steroids	Alive (6 yr)	126
**10**	M	61	CLL/SLL	Bone marrow	Anemia, weakness	NS+AKI	FC	Alive (3 yr)	137
**11**	M	68	CLL/SLL	Bone marrow	Anemia, lymphadenectasis	NS+AKI	Steroids	Died (4 mo)	NA
**12**	M	62	CLL/SLL	Bone marrow	Anemia, leucopenia	AKI	CTX	Alive (1 yr)	350
**13**	M	47	DLBCL	Kidney	Fever, weakness	AKI	CHOP	Alive (1 yr)	109
**14**	M	56	DLBCL	Kidney	Anemia	AKI	CHOP	Died (2 yr)	NA
**15**	M	62	DLBCL	Lymph node	Anemia, lymphadenectasis	AKI	Steroids	Alive (8 yr)	156
**16**	M	37	DLBCL	Ileum	Fever, diarrhea	proteinuria	CHOP	Died (2 yr)	NA
**17**	M	53	LPL	Lymph node	Anemia, lymphadenectasis	proteinuria	Steroids	Alive (2 yr)	89
**18**	M	68	LPL	Bone marrow	Fever, Anemia, skin rash	proteinuria	CTX	Died (1 yr)	NA
**19**	M	65	MCL	Lymph node	Anemia, lymphadenectasis	NS+AKI	CHOP	Alive (1 yr)	101
**20**	M	52	MALT	Stoma mucosa	Anemia, stomachache	NS+AKI	R+Steroids	Alive (1 yr)	249

CLL/SLL: chronic lymphocytic leukemia/small lymphocytic lymphoma, DLBCL: diffuse larger B-cell lymphoma, T/NK:T/NK cell lymphoma, TCL: T-cell lymphoma, MALT: mucosa-associated lymphoid tissue lymphoma, MCL: mantle cell lymphoma, LPL: lymphoplasmacytic lymphoma, NS: nephritic syndrome,

AKI: acute kidney injury, RPGN: rapidly progressive glomerulonephritis, R: radiotherapy, NA: not available.

CHOP: cyclophosphamide, hydroxydaunomycin, oncovin, prednisone. CTX: cyclophosphamide.

EPOCH:Etoposide, prednisone, oncovin, cyclophosphamide, hydroxydaunomycin, FC: fludarabine, cyclophosphamide

Of the 20 patients with NHL, 16 (80%) had B-cell neoplasms, including chronic lymphocytic leukemia/small lymphocytic lymphoma (CLL/SLL, n = 8), diffuse large B-cell lymphoma (DLBCL, n = 4), lymphoplasmacytic lymphoma (LPL, n = 2), mucosa-associated lymphoid tissue lymphoma (MALT, n = 1) and mantle cell lymphoma (MCL, n = 1). Four patients (20%) had mature T/NK-cell neoplasms, including extra nodal NK/T-cell lymphoma, nasal type T/NK cell lymphoma (n = 2), cutaneous T-cell lymphoma (n = 1) and adult T-cell lymphoma (n = 1).

All patients presented with proteinuria, and 15 patients (75%) had impaired renal function(eGFR<60 ml/min). The mean serum creatinine level was 228±181 µmol/l (range 50 to 752 µmol/L). Microscopic hematuria was found in 15 patients (75%), and gross hematuria in 4 patients (20%). Renal symptoms included nephrotic syndrome in 6 patients, while accompanied by AKI in 4 patients; rapidly progressive glomerulonephritis (RPGN) in 5 patients; AKI in 5 patients and mild proteinuria/microscopic hematuria in the remaining 4 patients (see Table 2).

**Table 2:** Summary of clinical data in patients with NHL and renal involvement

Patient	Hb (g/L)	WBC (*10^9^/L)	Scr (µmol/l)	eGFR (ml/min)	albumin (g/L)	globulin (g/L)	Urine protein (g/d)	Urine RBC (10^4^/ml)	Urine NAG (U/g·cr)	Urine RBP (mg/L)	Renal size (mm/mm)	Antibody or Cryo	SIFE
**1**	70	6.6	187	44.8	33	31.3	2.72	2500	40.7	65.5	115/109	N	N
**2**	90	4.1	61	105	28.2	35.3	3.5	95	31.2	1.51	103/101	ANA PR3-ANCA	N
**3**	65	4.5	100	52.7	32.6	28.8	1.58	N	47.6	1.32	104/100	N	N
**4**	147	5.2	95	85.9	20.4	16.4	5.37	15	39.4	1.13	109/105	N	N
**5**	59	2.8	543	13	38.4	38.7	1.3	2000	40.2	7.78	106/102	PR3-ANCA	N
**6**	66	4.0	151	32.8	36.4	29.9	3.02	1850	94	1.88	99/98	ANA	N
**7**	91	5.1	338	15.6	36.5	26.3	1.41	1250	26.9	10.2	107/103	A-GBM	N
**8**	99	10.1	477	12.6	31.6	45.7	2.75	3000	35.5	10.2	127/122	Cryo	N
**9**	87	25.3	188	32.9	38.9	27.8	4.24	750	83.2	0.43	100/98	N	N
**10**	99	11.4	320	18.3	23.6	29.9	10.2	15	48.2	31.7	97/96	N	IgM, k
**11**	111	7.3	235	25.6	17.1	29.1	14.9	570	75.4	53.4	94/96	N	IgM, k
**12**	89	3.3	392	17.8	27.7	17.7	0.71	N	25.2	4.5	123/120	Cryo	IgM, k
**13**	135	8.8	158	46.3	44.2	29.7	0.39	N	20.2	10	167/146	N	N
**14**	98	5.6	166	37.2	40.6	27.4	0.84	N	32.4	3.44	114/110	N	N
**15**	113	4.2	168	34.2	38.3	15.5	1.24	47	21.1	0.13	103/101	N	N
**16**	107	10.6	77	103	36	18	0.68	50	25.7	0.1	106/101	N	N
**17**	109	4.5	74	103	34	40.3	0.41	N	26.2	0.93	129/120	N	IgM, k
**18**	78	9.4	50	95.8	27.2	18.8	0.7	230	82.3	0.84	111/110	Cryo	N
**19**	80	13.8	164	34.8	28.4	28.6	6.92	260	25	15.5	112/115	N	N
**20**	106	3.7	263	36.5	32.3	18.7	7.96	1100	119	11.9	112/106	N	IgM, k

Hb, hemoglobin; WBC, white blood count; BUN, blood urea nitrogen; SCr, serum creatinine; eGFR: estimated Glomerular Filtration Rate

NAG, N-acetyl-pD glucosaminidase enzyme, RBP: retinol-binding protein (RBP). ANA: antinuclear antibody, C-ANCA: antineutrophil cytoplasmic antibody, PR3-ANCA: anti-proteinase 3 ANCA, A-GBM: anti-glomerular basement membrane antibody, Cryo: cryoglobulinemia; SIFE: serum immunofixation electrophoresis, N negative

Eighteen patients (90%) had anemia (5.9 to 11.3 g/dl), five had leukocytosis, three had leucopenia and three had thrombopenia. Two patients were positive for ANA, two were positive for cytoplasmic antineutrophil cytoplasmic antibodies (C-ANCA) and anti-proteinase 3 ANCA (PR3-ANCA) and one was positive for anti-glomerular basement membrane antibody (anti-GBM). IgM (kappa) monoclonal protein was detected in five patients by immunofixation electrophoresis. Cryoglobulinemia was also identified in three patients (see Table 2).

### Histological findings

**Figure 1:**
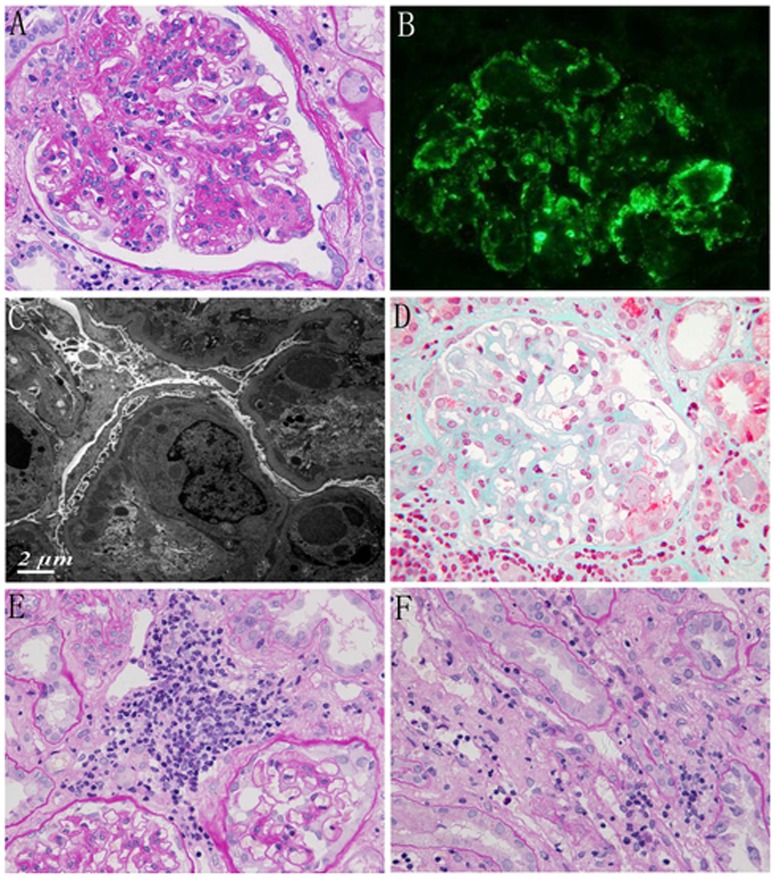
Representative renal histological change in patients with NHL. A Light microscopy showed increased cellularity in glomeruli and the accentuation of the lobular architecture (patient 9 PAS×400). B Immunofluorescence showed granular staining in a lobular pattern for IgG (patient 9 IF×400). C Electron microscopy showed fine subendothelial electron-dense deposits (patient 9 EM). D Glomeruli with focal segmental necrosis and lymphoid cell infiltration around glomeruli (patient 8 Masson×400). E Glomeruli without pathological abnormalities visualized by LM with lymphocyte-like cell infiltration in the interstitial tissue (patient 5 PAS×200). F Mononuclear cell infiltration in the peritubular capillaries (patient 5 PAS×400).

The histological findings of 20 patients are summarized in Table 3. Membranoproliferative glomerulonephritis -like patterns was found in seven patients with B-cell lymphoma. Light microscopy showed increased cellularity in all glomeruli and accentuation of the lobular architecture (Fig. 1 A). Silver staining showed double contours within the capillary loops. Capillary loops contained infiltrating leucocytes (more than 10 cells). Immunofluorescence studies revealed granular staining in a lobular pattern for IgG, IgM or C3 in five patients (Fig. 1 B). IgG was undetectable, and only IgM and C3 were present in mesangial area in case 6 and 18. The interstitium was diffusely or focally infiltrated by mononuclear cells. Electron microscopy showed fine subendothelial and mesangial electron-dense deposits together with frequent double-contoured appearance (Fig. 1 C).

**Table 3:** Renal biopsy findings

	Glomerular Disease	Infiltrating cells		Immunofluorescence							Immunoperoxidase		
		Glomerular	PTC	IgG	IgA	IgM	C3	C1q	κ	λ	CD20(n/mm^2^)	CD68(n/mm^2^)	
**1**	Crescent GN	>5	Y	-	-	-	-	-	-	-	NA	192	
**2**	Crescent GN	2–3	Y	-	-	-	-	-	-	-	NA	NA	
**3**	NSPA	2–3	No	+	-	+	-	-	-	-	96	NA	
**4**	MCD	2–3	Y	-	-	-	-	-	-	-	Multifocal	152	
**5**	NSPA	>3	Y	++	-	-	++	++	-	+	NA	624	
**6**	MPGN	>10	Y	-	-	+	+	+	-	-	Diffuse	548	
**7**	Crescent GN	>5	Y	++	-	-	-	-	-	+	Multifocal	Diffuse	
**8**	Crescent GN	>5	Y	++	+	+	++	++	+	+	Multifocal	592	
**9**	MPGN	>10	Y	++	+	++	++	+	++	+	Multifocal	152	
**10**	MCD	3–5	Y	-	-	-	-	-	-	-	184	872	
**11**	MCD	3–5	Y	-	-	-	-	-	-	-	72	356	
**12**	ICMDD	3–5	Y	-	-	++	-	-	++	-	Diffuse	140	
**13**	No glomerular	ND	Y	-	-	-	-	-	-	-	192	1192	
**14**	Lymphoma	ND	Y	-	+	+	-	-	-	-	16	848	
**15**	MPGN	>10	Y	++	+	++	+	++	+	++	NA	NA	
**16**	MPGN	>10	No	++	++	-	++	-	+	+	144	NA	
**17**	NSPA	3–5	Y	-	-	-	-	-	-	-	Multifocal	312	
**18**	MPGN	>5	Y	-	++	-	++	-	+	-	Focal	268	
**19**	MPGN	>10	No	++	-	-	++	++	-	+	180	420	
**20**	MPGN	>10	No	++	++	-	++	-	++	+	NA	312	

MPGN: Membranoproliferative glomerulonephritis. FSNGN: Focal segmental necrotizing glomerulonephritis. PTC:

ICMDD: Intracapillary monoclonal IgM deposits. Lymphoma: Intraglomerular lymphoma. MCD: Minimal-change disease NSPA:No specific pathologic abnormalities; NA: not available. Immunofluorescence: -, negative; +, mild; ++, intense. λ: λ light chain; κ: κ light chain.

Crescent glomerulonephritis was demonstrated in 4 patients, two patients had T/NK cell lymphoma, and the remaining two had CLL/SLL. No evidence of immune complex deposition was found in case1, 2; and serum PR3-ANCA was positive in case 2. IgG line deposits along the GBM and anti-GBM antibody was positive in case 7. Cellular crescents and focal necrotizing capillary by LM and IgG, IgM, IgA and C3 deposits in the mesangial and capillary were confirmed in case 8. In addition to the glomerular lesions, mononuclear cell infiltration was observed in the peritubular capillaries (PTCs) in all 4 patients.

Three patients (case 4,10,11) had minimal change in the glomeruli observed by light microscopy and no immune complex deposition by IF assay, but ultrastructural examination of these glomeruli revealed extensive effacement of the foot processes characteristic of minimal-change disease (MCD). The other three patients(case 3,5,17) revealed glomeruli without specific pathological abnormalities, ultrastructural examination revealed only segmental effacement of the foot processes, and the IF assay showed staining for IgG and IgM deposits in the glomeruli in two patients(case 3,5). Mononuclear cell infiltration was also observed in the PTCs in five patients (Fig. 1 E, F).

**Figure 2:**
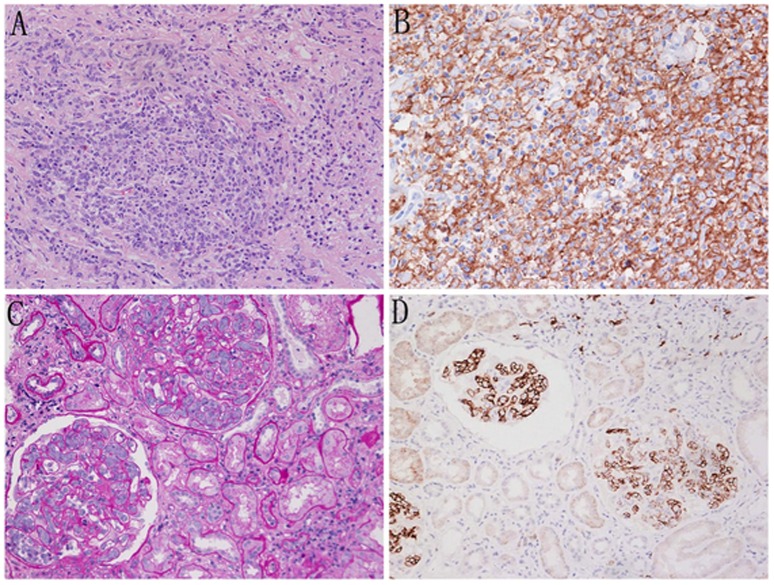
Diffuse infiltration into the renal parenchyma and intraglomerular lymphoma in DLBCL. A. Accumulation of large atypical lymphoid cells was observed in renal specimens (patient 13 PAS×400). B. Immunohistochemical staining showing strong staining for CD20 in the lymphoid cells (patient 13 IH×400). C. Glomerular capillary lumina containing atypical large cells in the enlarged glomeruli (patient 14 PAS ×400). D Immunohistochemistry showing strong staining for CD20 in lymphoid cells localized within the glomerular capillary lumina (patient 14 IH×400).

Primary diffuse large B-cell lymphoma of the kidney was found in case 13. Severe cell infiltration throughout the cortex and marked destruction of both the glomeruli and the tubular structure, with only two ruined glomeruli were observed. The accumulation of large atypical lymphoid cells with hyperchromatic nuclei was observed in renal specimens under high-power magnification (Fig. 2A). IF did not reveal any glomerular immune deposits. The lymphoid cell were positive for CD20 (+++) (Fig. 2B) and CD79a (+) but negative for CD3, CD43 and CD138.

Intraglomerular large B-cell lymphoma was identified in case 14. Lymphoid cells were present only within the lumina of the glomerular capillaries and not within the interstitium (Fig 2C). Large pleomorphic lymphoid cells often occluded the glomerular capillaries. The lymphoid cells had large vesicular nuclei. In the IF analysis of frozen sections, the lymphoid cells were CD45^+^, CD3^−^ and CD20^3+^ (Fig 2D).

**Figure 3:**
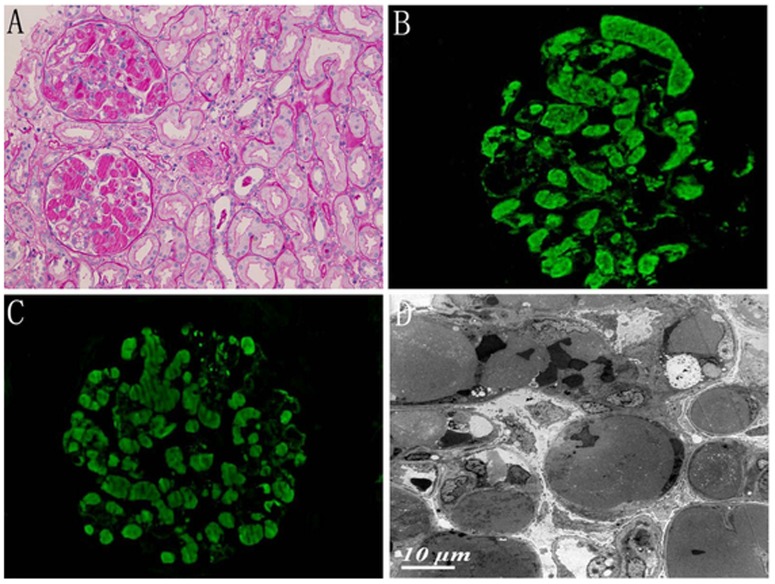
Intracapillary monoclonal deposition disease in an NHL patient (patient 12). A PAS-positive intracapillary thrombi in glomerulus (PAS, ×200). B Immunofluorescence showed bright staining of the intraglomerular thrombi for IgM. C Immunofluorescence showed bright staining of the intraglomerular thrombi for κ LC.D Electron microscopy showed that the glomerular capillary lumen was filled with granular electron-dense material. No organized structure was observed.

Intracapillary monoclonal IgM deposits (ICMDD) was observed in case 12. The most striking glomerular lesions consisted of periodic acid Schiff-positive intracapillary deposits that were variable in size and distribution (Fig. 3A). In the IF assay, all of the deposits were strongly stained for IgM and the κ light chain (Fig. 3B, C). EM revealed large intracapillary deposits that dilated the capillary lumen. No organized structure was observed (Fig. 3D).

The monomorphic lymphocytes infiltrates were patchy in 7 cases, diffuse in case 13 (Table 3), renal tubules were separated and atrophic or destroyed as a result of interstitial infiltrates. Staining with various immunohistochemical markers revealed that the infiltrating lymphocyte cells were positive for predominance of CD20, while CD68^+^ cells within the infiltration (Table 3), which is consistent with B-cell lymphoma.

### Outcome

During follow-up, two patients were lost, nine patients died. Among those who died, three patients with T/NK cell lymphoma died of infection after they received specific chemotherapy; the remaining six patients died as a direct or indirect result of the lymphoma during the first 2 years after NHL diagnosis. In these nine patients, four patients had AKI, four patients had CKD in last record. At the end of the follow-up period (6-96 months, average 35 months), three patients had normal serum creatinine, five patients had renal function stable or improved after chemotherapy, and one patient required permanent hemodialysis. (See Table 1).

## Discussion

In this study, we confirmed that a wide spectrum of NHL is associated with renal injury, particularly with B-cell lymphocytic type NHL. CLL/SLL was the most common type of lymphoma associated with renal involvement (40%), followed by DLBCL and NK/T-cell lymphoma (20%). The profile of the NHL reported in the literature is similar to that observed our study. Kowalewska et al. [6] found a similar pattern of distribution; among 18 patients with neoplasms and kidney injury, the most common type of lymphoma was CLL/SLL, followed by DLBCL. However, Sun et al. [10] showed that the most common subtype of lymphoma was DLBCL (36.2%) in China, followed by extranodal NK/T-cell lymphoma, nasal type (11.0%); CLL/SLL accounted for only 3.7% of all lymphoid neoplasm. This finding suggests that renal involvement may be commonly associated with CLL/SLL. The pathogenesis of CLL/SLL-associated glomerulonephritis is not well understood. Notably, four of eight patients with CLL/SLL had serum positive for ANA or ANCA, three had M-component and two had cryoglobulinemia in their sera. Therefore, glomerulonephritis may also be related to the production of cryoglobulins and/or immunoglobulins (paraprotein production) by secretory B-cell clones in patients with CLL/SLL.

In our series, the most common presentations of renal involvement in NHL patients were renal impairment and proteinuria. All patients presented with proteinuria, and 75% had impaired renal function; 30% had nephrotic syndrome and 25% had RPGN. It is difficult to distinguish lymphoma-associated glomerulonephritis from primary kidney disease in the clinic. Interestingly, at the time of the renal biopsy, only 10% patients in our series had an established diagnosis of current NHL. Therefore, renal biopsy may be suggested as the diagnostic method of choice.

The most common pattern of glomerular involvement in our series was glomerulonephritis with MPGN-like patterns. All seven cases of MPGN-like patterns were associated with B-cell neoplasm. Among these patients, three were positive for serum ANA, M-component or cryoglobulinemia. IgG deposition was not observed in two patients, which is different from typical MPGN. Moulin et al. [11] retrospectively analyzed 13 patients with glomerulonephritis and chronic lymphocytic leukemia (CLL) or B-cell lymphoma; MPGN was observed in eight patients, five patients had cryoglobulinemia and two patients had circulating M-component. Therefore, MPGN- like patterns associated with NHL may directly result from the lymphoma through a paraprotein deposition process that results in cryoglobulinemia or monoclonal immunoglobulin deposition disease, or it may be induced indirectly through immune-mediated mechanisms.

Four patients had crescent glomerulonephritis in our series; one patient with PR3-ANCA, one with anti-GBM antibody. Pauci-immune crescentic glomerulonephritis associated with NHL and ANCA has also been reported by Henriksen and Dussol [12]–[13]. However, we describe a previously unreported unique case of anti-GBM glomerulonephritis in a patient with CLL/SLL. Given that NHL patients have defective T-cell colony-forming capacity and T-cell regulatory function, the simultaneous presence of NHL, anti-GBM and crescentic glomerulonephritis may not be a coincidence.

Minimal-change nephrotic syndrome is described as a paraneoplastic manifestation of classical Hodgkin's lymphoma (HL) that occurs in 0.4% of Hodgkin's lymphoma patients but is a rare complication of NHL [14]–[15]. In our series, three patients presented with nephritic syndrome, and showed MCD by EM. Increased cytokine levels caused by T- or B-cell lymphoma cytokine-induced altered glomerular permeability and podocyte injury may be responsible for nephrotic syndrome [15]. In these patients, mononuclear cell infiltration into the PTC and lymphocytic infiltration of the tubule interstitium were also observed. It is not clear why patients with NHL associated renal injury have mononuclear cell infiltration into the PTC. One possibility would be that NHL induces the release of proinflammatory molecules, which induce infiltrating cells infiltration. These results suggest that the immune processes involved in the pathogenesis of NHL-associated MCD are different from those of idiopathic MCD.

ICMDD was defined as PAS-positive intracapillary thrombi in glomeruli that were sufficiently voluminous to occlude the capillary lumens and thrombi positive for IgM and either κ or λ LC by IF. This type of renal lesion was first reported in Waldenstrom macroglobulinemia (WM). However, Audard et al. [16] reported that only three of five patients with ICMDD had WM, whereas the two other patients had an IgM-related disorder and a B-cell lymphoma. In our study, patients with intraglomerular thrombi were found to have circulating monoclonal IgM and κ LC. Cellular proliferation in the glomeruli was absent or mild; no organized microtubular structures were visualized by EM, which suggests a diagnosis of ICMDD rather than cryoglobulinemic glomerulonephritis. Therefore, ICMDD may be applied to IgM-secreting B-cell lymphoma. In addition, κ and/or λ light chain staining was positive by immunofluorescence in 10 patients in our study. However, no linear deposits of κ or λ light chain staining within GBM and TBM was observed, which was the characteristic for light chain deposition disease (LCDD).

Renal involvement is rare in patients with diffuse large B-cell lymphoma (DLBCL). Villa et al. [17] identified renal involvement in only 2% (55/2656) of patients with DLBCL at the time of diagnosis. In the present study, computed tomography (CT) revealed markedly enlarged kidneys bilaterally in case 13, and the renal parenchyma was massively infiltrated by CD20^+^ lymphoid cells; the lymphoma was classified as diffuse large B-cell lymphoma. Intravascular large B-cell lymphoma (IVLBCL) is a very rare subtype of NHL lymphoma. To the best of our knowledge, no more than 20 cases have been published in the literature [18]–[19]. The patients with intraglomerular large B-cell lymphoma may present with proteinuria, renal dysfunction, which may clinically mimic primary glomerulonephritis. The glomerular lesion may appear confusingly like endocapillary proliferative glomerulonephritis; only immunocytochemical characteristics can reveal the true lymphoid cells [20]. In such cases, kidney biopsy is the only way to establish the correct diagnosis.

In our study, 40% patients showed lymphomatous infiltration of the interstitium. All of these patients had B-cell lymphoma, and seven of eight presented with AKI. Lymphocytic infiltrations of the renal tubulointerstitium and AKI have been reported previously [2], [6]. The mechanism of AKI due to lymphomatous infiltration of the kidneys is unknown; infiltrate may destruct of the normal renal architecture and cause intrarenal obstruction. The aggregates of inflammatory cells such as CD68+ cells can also be seen in these patients, which suggest to inflammatory response to lymphomatous infiltration. A monotonous appearance and aggregates of infiltrative pattern of the lymphomatous cell population in kidney should prompt the examiner to perform an additional workup to rule out or confirm lymphoma.

The differentiation between a primary glomerular disease and lymphoma-associated glomerulonephritis is difficult. Therefore, we need to implement screening for the underlying hematological malignancy if a patient presents with (1) severe anemia, leukocytosis and/or thrombopenia; (2) enlarged lymph nodes and/or enlarged kidneys; (3) mononuclear cells in the PTC or monomorphic lymphoid cells infiltration in renal parenchyma. If a patient has one or more of the above conditions, it is imperative that the patient undergo a full workup for lymphoma, which should include lymph node biopsy, bone marrow aspiration and biopsy, serum immunofixation studies and cryoglobulinemia detection. If these tests are positive, then a series of immunohistochemical staining studies is warranted to determine whether the kidney injury is caused by underlying lymphoma.

In conclusion, we confirmed that a wide spectrum of renal pathological findings can be observed in patients with NHL, and NHL may be first diagnosed by renal biopsies for evaluation of kidney injury or proteinuria. CLL/SLL were the most common types of NHL associated with renal injury, and the most common pattern of glomerular lesion was MPGN-like pattern. Renal biopsy is necessary to establish the underlying cause of renal involvement in NHL.
